# How Should Non‐Prescribed Use of Gabapentinoids be Reduced in Psychiatric Practice?

**DOI:** 10.1002/hup.70010

**Published:** 2025-07-12

**Authors:** Giada Lorenzi, David S. Baldwin

**Affiliations:** ^1^ Faculty of Medicine University of Pisa Pisa Italy; ^2^ Clinical and Experimental Sciences University of Southampton Southampton UK

Gabapentinoids are a group of medications used for treating patients with a limited range of neurological or psychiatric conditions: some (gabapentin and pregabalin) have entered widespread clinical use, due to their analgesic, anticonvulsant and anxiolytic effects. Licenced indications for these drugs vary across countries: for example, within the United Kingdom (UK), gabapentin does not have a market authorisation for treating patients with psychiatric disorders, although it is licenced for treatment of focal seizures, peripheral neuropathic pain, menopausal symptoms in women with breast cancer, oscillopsia and spasticity in multiple sclerosis, and muscular symptoms in motor neuron disease. By contrast, pregabalin has a license for generalized anxiety disorder (GAD), and for treating patients with peripheral or central neuropathic pain, and as an adjunctive therapy in patients with focal seizures: however, it is not licenced for GAD within the United States. Since introduction, gabapentin and pregabalin have become often prescribed outside the terms of their market authorisations in many countries: at least half of all gabapentinoid prescriptions in the UK may be ‘off‐label’ [Montastruc et al. 2018], and concerns regarding widespread non‐prescribed use (with some fatalities) led to their reclassification as Class C controlled substances with accompanying regulations regarding prescriptions, in April 2019. Since this reclassification, further data on potential hazards associated with non‐prescribed use of gabapentinoids have accumulated [Baldwin and Masdrakis 2022].

Findings from epidemiological studies and systematic reviews indicate that the prevalence of non‐prescribed gabapentinoid use is not insubstantial: for example, in populations without a history of substance misuse the prevalence of non‐prescribed use of pregabalin may lie between 0.5% and 8.5% among those dispensed the drug [Schjerning et al. 2016]. A range of risk factors for non‐prescribed use for gabapentinoids have been identified: individuals with a history of substance use disorders are at greater risk, particularly those with a history of opiate use or poly‐substance use. Other possible risk factors for non‐prescribed use include younger age, male sex, a diagnosis of anxiety, access to multiple prescribers, and physical illness including cancer, multiple sclerosis and neuropathy [Driot et al. 2019].

There are multiple hazards associated with non‐prescribed use of gabapentinoids [Baldwin and Masdrakis 2022]. Non‐prescribed ‘overuse’ of gabapentin is associated with increased risks of all‐cause and drug‐related hospitalisation, particularly if combined with opioids, and pregabalin prescriptions in patients undergoing opioid maintenance therapy may increase all‐cause mortality: increased risk of death may result from respiratory depression, prolonged gastrointestinal transit increasing gabapentin concentration, and the delayed onset of effect of gabapentinoids when compared to injected opioids. Gabapentinoid users may have increased risks of ‘suicidal behaviour’ [Yuen et al. 2025] unintentional overdoses, road traffic accidents, legal offences, and head/body injuries [Molero et al. 2019].

Non‐prescribed use may also be associated with dependence and withdrawal syndromes, but these are not well characterised. Possible withdrawal symptoms include anxiety, insomnia, depression, agitation, irritability, ‘paranoia’, psychosis, suicidal thoughts, headache, joint and muscle pains, lethargy, shivering, sweating, tachycardia, disorientation and seizures; and possible neonatal withdrawal syndromes have also been described. The proportion of patients who experience withdrawal symptoms is not established, but agitation, confusion and disorientation occur in approximately half of cases, following abrupt discontinuation of gabapentin [Ashworth et al. 2023]: withdrawal symptoms can occur even after only short treatment courses. Withdrawal symptoms typically have their onset within 24–48 h of discontinuation, although may be delayed for up to seven days.

Clinical management of benzodiazepine dependence is founded on prevention, early recognition, and combining gradual dosage reduction with simple psychological supportive measures [Baldwin 2022]: although anxiolytic, gabapentinoids have a differing mechanism of action to benzodiazepines and it cannot be assumed that non‐prescribed use of gabapentinoids is managed optimally in a similar way. Over‐arching principles of clinical management of non‐prescribed use of gabapentinoids necessarily include awareness of potential risks: mental health professionals should not regard gabapentinoids as benign anxiolytics, and alternatives such as psychotherapeutic approaches and differing medication classes should be considered. Clinicians should reflect on potential hazards when contemplating prescription of gabapentinoids, and act to prevent non‐prescribed use through careful risk assessment, counselling regarding potential risks associated with treatment prior to the first prescription, cautious prescribing, and concerted monitoring for signs of misuse: and be vigilant for indicators such as requests for higher dosages, obtaining prescriptions from multiple providers, and reports of lost medications.

There is currently much uncertainty about the rate by which gabapentinoids should be withdrawn, or whether concomitantly prescribed medicines may ease the process of withdrawal [Parsons 2018; Anderson et al. 2023]. This uncertainty needs to be addressed through research, algorithms and audit. Steady tapering of dosage has been recommended, but the evidence to support prolonged ‘hyperbolic tapering’ of gabapentinoids is extremely limited. Reports of the concomitant use of benzodiazepines have produced conflicting findings, and switching between gabapentinoids seems ineffective. Case reports have suggested some benefit of antipsychotic drugs in managing delirium associated with withdrawal. In the current absence of definitive evidence, it makes sense to warn patients not to stop treatment abruptly, encourage patients to strengthen their support network when contemplating withdrawal from treatment, reduce dosage at a rate agreed with the patient, consider switching to liquid formulations to facilitate dosage tapering, and review progress regularly during the process of withdrawal.

## Conflicts of Interest

D.S.B. is Editor‐in‐Chief of *Human Psychopharmacology*. G.L. declares no conflicts of interest.

## Data Availability

The authors have nothing to report.
